# The Journal’s Own Page

**Published:** 1897-02

**Authors:** 


### THE JOURNAL'S OWN PAGE.

---

*The Red Back Gains Permanent Friends.*—Dr. J. Enos Butler, Eastland, Tex., in renewing his subscription, writes: "I shall discontinue a number of the journals I have been taking; but I *must have* the "Red Back!" * * I cannot *afford to do without* the TEXAS MEDICAL JOURNAL once a month, and I think that every Texas physician who would keep up with the progress of the medical profession should subscribe for it; it is our *home* journal."

